# Comparison of the Combined versus Conventional Apgar Scores in Predicting Adverse Neonatal Outcomes

**DOI:** 10.1371/journal.pone.0149464

**Published:** 2016-02-12

**Authors:** Hosein Dalili, Mahdi Sheikh, Amir Kamal Hardani, Firouzeh Nili, Mamak Shariat, Fatemeh Nayeri

**Affiliations:** 1 Breastfeeding Research Center, Vali-asr Hospital, Tehran University of Medical Sciences, Tehran, Iran; 2 Maternal, Fetal and Neonatal Research Center, Vali-asr Hospital, Tehran University of Medical Sciences, Tehran, Iran; Centre Hospitalier Universitaire Vaudois, FRANCE

## Abstract

**Objectives:**

Assessing the value of the Combined-Apgar score in predicting neonatal mortality and morbidity compared to the Conventional-Apgar.

**Methods:**

This prospective cohort study evaluated 942 neonates (166 very preterm, 233 near term, and 543 term) admitted to a tertiary referral hospital. At 1- and 5-minutes after delivery, the Conventional and Combined Apgar scores were recorded. The neonates were followed, and the following information was recorded: the occurrence of severe hyperbilirubinemia requiring medical intervention, the requirement for mechanical ventilation, the occurrence of intraventricular hemorrhage (IVH), and neonatal mortality.

**Results:**

Before adjusting for the potential confounders, a low Conventional (<7) or Combined (<10) Apgar score at 5-minutes was associated with adverse neonatal outcomes. However, after adjustment for the gestational age, birth weight and the requirement for neonatal resuscitation in the delivery room, a depressed 5-minute Conventional-Apgar score lost its significant associations with all the measured adverse outcomes; after the adjustments, a low 5-minute Combined-Apgar score remained significantly associated with the requirement for mechanical ventilation (OR,18.61; 95%CI,6.75–51.29), IVH (OR,4.8; 95%CI,1.91–12.01), and neonatal mortality (OR,20.22; 95%CI,4.22–96.88). Additionally, using Receiver Operating Characteristics (ROC) curves, the area under the curve was higher for the Combined-Apgar than the Conventional-Apgar for the prediction of neonatal mortality and the measured morbidities among all the admitted neonates and their gestational age subgroups.

**Conclusions:**

The newly proposed Combined-Apgar score can be a good predictor of neonatal mortality and morbidity in the admitted neonates, regardless of their gestational age and resuscitation status. It is also superior to the Conventional-Apgar in predicting adverse neonatal outcomes in very preterm, near term and term neonates.

## Introduction

The Apgar score is the oldest and most commonly used assessment tool for the evaluation of the newborn in the delivery room; first described in 1950s [1], it soon achieved a legendary status as the outcome measure for countless studies.[2–4] However, this use has long been a matter of controversy; since the early 1990s, many researchers criticized the use of the Apgar score for the prediction of neonatal outcomes due to its serious limitations, especially in premature and resuscitated neonates. Therefore, it was suggested to use Apgar scores with caution, while the search continued for an alternative that could be used in assessing all newborns.[2,5–8]

The American College of Obstetricians and Gynecologists (ACOG) and the American Academy of Pediatrics (AAP) in the committee opinion on Apgar score determined that the Apgar score should not be used alone to predict adverse neonatal outcomes, due to several limitations of the Apgar score, including that it is influenced by the neonate’s maturity and the medical interventions in the delivery room.[6,9] Therefore, the Specified-Apgar and later, the Expanded-Apgar scores, were suggested to allow an assessment of the newborn's condition independent of gestational age and interventions.[9,10,11] Despite these attempts, there was still a need for a more comprehensive and precise scoring system to predict the occurrence of adverse neonatal outcomes. The Combined-Apgar score was therefore proposed by Rudiger et al., which combines both the Specified and the Expanded Apgar scores, thereby allowing a more detailed description of the neonate's postnatal condition.[12]

Currently, there is no accepted standard for evaluating the newborns under clinical conditions in the delivery room, especially the preterm and resuscitated newborns.[8,9] To date, only two published studies in the English literature with promising results have evaluated the Combined-Apgar score;[13,14] Rudiger et al. evaluated the Combined-Apgar score in the prediction of neonatal mortality and morbidity among very preterm neonates,[13] and Dalili et al. evaluated the Combined-Apgar score in the prediction of asphyxia and early neurologic outcomes in asphyxiated neonates.[14] Both studies concluded that the Combined-Apgar score was superior to the Conventional-Apgar in predicting adverse neonatal outcomes and concluded that more studies are required in this field.[13,14] The value of the Combined-Apgar score in predicting adverse outcomes among near term and term neonates and among the admitted neonates in general, is still unknown.

We therefore conducted this study to evaluate the value and applicability of the Combined-Apgar score among the admitted neonates in general, regardless of their gestational age or resuscitation status and among different subgroups of the admitted neonates (term, near term and very preterm) and to compare the predictive values of the Combined-Apgar to the Conventional-Apgar score in predicting neonatal morbidity and mortality.

## Materials and Methods

### Study population and study design

This prospective cohort study was conducted on the newborns admitted between September 2012 and February 2015 to a tertiary referral hospital with an annual birth rate of 2200 births. Inclusion criteria were live birth at a gestational age of more than 25 weeks, birth within the study center, and the requirement for hospital admission. The exclusion criteria were a gestational age at or below 25 weeks, birth outside of the study center, major congenital anomalies, death in the delivery room, and missing parental informed consent. After obtaining a written informed consent from the parents, a total of 942 neonates born at the gestational ages of 26–40 weeks participated in the study, which was approved by the Research Deputy and the Ethics Committee of our institute.

### Data and specimen collection

The gestational age at birth was calculated based on ultrasound imaging. Neonates who were born before 32 weeks of gestation were considered very preterm, those who were born at a gestational age between 32 and 37 gestational weeks were considered near term, and neonates who were born at ≥37 gestational weeks were considered term. The type of delivery, birth weight, and neonatal gender were recorded for each participant. After the neonate was delivered, the 1- and 5 minute Conventional and Combined Apgar scores were recorded in the delivery room by educated physicians according to Table 1 and Table 2.[1,12]

**Table 1:** The Conventional-Apgar scoring system as introduced by Virginia Apgar in 1953.

Sign	The Score		
	0	1	2
**Heart rate**	Absent	Less than 100	More than 100
**Respiration**	Absent	Slow, irregular	Good, Crying
**Muscle tone**	Limp	Some flexion	Active motion
**Reflex Irritability**	No response	Grimace	Cough, sneeze, cry
**Color**	Blue or pale	Pink body, blue extremities	Completely pink

**Table 2:** The Combined-Apgar scoring system, that consists of the Expanded and Specified Apgar scoring systems as introduced by Rudiger et al in 2012 [12].

		Minutes		
		1	5	10
C	Continuous Positive Airway Pressure (a)			
O	Oxygen			
M-B	Mask and Bag Ventilation (b)			
I	Intubation and Ventilation			
N	Neonatal Chest Compression			
E	Exogenous Surfactant			
D	Drugs			
	**Sum of Expanded Apgar**			
	**Scoring Each Item:**			
	0 = Intervention was performed; 1 = No intervention was performed			
	(a): score 0 if “Mask and Bag” or “Intubation and Ventilation” is score 0			
	(b): score 0 if “Intubation and Ventilation” is scored 0			
**A**	**Appearance (Skin Color)**			
	2 = Completely pink			
	1 = Centrally pink with acrocyanosis			
	0 = Centrally blue or pale			
**P**	**Pulse (HR)**			
	2 = > 100 beats per minute			
	1 = < 100 beats per minute			
	0 = No heart beat			
**G**	**Grimacing (Reflex)**			
	1 = Reduced for gestational age			
	2 = Appropriate for gestational age			
	0 = No reflex response			
**A**	**Activity (Muscle Tone)**			
	2 = Appropriate for gestational age			
	1 = Reduced for gestational age			
	0 = No reflex response			
**R**	**Respiration (Chest Movement)**			
	2 = Regular chest movement			
	1 = Small of irregular chest movement			
	0 = No chest movement			
	**Sum of Specified Apgar**			
	**Total (Sum of Expanded + Specified)**			

The Conventional-Apgar score ranges from 0–10 and the Combined-Apgar score ranges from 0–17. A Conventional-Apgar score <7 and a Combined Apgar score <10 were considered depressed. The cutoff points were chosen based on the ACOG and AAP definitions of the abnormal Apgar score as stated in their committee opinions on Apgar score [9] and also on the available literature that assessed the predictive value of the Combined Apgar score in specific populations.[13,14]

The physicians were educated in the delivery room before the beginning of the study to assure the consistency and avoid interpersonal biases of Apgar scores calculations. Then, the neonates were followed by a neonatologist until discharge, and the following information was recorded: the occurrence of severe neonatal jaundice requiring intensive phototherapy and/or exchange transfusion, the requirement for mechanical ventilation, the duration of total hospital admission and neonatal mortality. In addition, intracranial ultrasound imaging was performed on all the neonates during the first postnatal week, to detect the occurrence of intraventricular hemorrhage (IVH).

### Statistical analysis

All statistical analyses were performed using SPSS statistical software (*version 22*: *IBM*, Chicago, IL). Chi-squared analysis and the Fisher's exact test were used to analyze the categorical and qualitative variables, whereas the independent-samples t-test and One-way ANOVA were used to analyze the numerical and quantitative variables. Multivariate logistic regression was used for adjusting the results and assessing their dependency, and Receiver Operating Characteristics (ROC) curves were used to assess the predictive values and area under the curves (AUC). The sample size was calculated for a power of 80% and an alpha error of 0.05; based on the neonatal mortality rate in our country and the reported relative risk for the low 5-minute Apgar score and infant death in the literature, almost 50 neonates were required in each study group to detect statistically significant differences in the mortality and morbidity rates between those with depressed Conventional/Combined Apgar vs. normal Apgar scores. The estimated odds ratios (ORs) with 95% confidence intervals (95% CIs) and P value <0.05 were used to evaluate the statistical significance of the associations and correlations between variables.

## Results

### Descriptive statistics

This prospective cohort study was conducted on 942 admitted neonates who were born at gestational ages >25 weeks. Table 3 summarizes the demographics of the studied subgroups (Table 3). In total, 166 neonates (17.6%) were very preterm, 233 (24.7%) were near term, and 543 neonates (57.6%) were term. Overall, 188 neonates (19.9%) required resuscitation in the delivery room, 179 (19%) had jaundice requiring intensive phototherapy and/or exchange transfusion, 62 (6%) required mechanical ventilation, IVH was detected in 47 neonates (5%), and mortality occurred in 24 (2.5%).

**Table 3:** Demographics of the study population.

Demographic Factors	Mean ± SD
Gestational Age (weeks)	
Very preterm	29.2 ± 2.1
Near term and Term	37.4 ± 2.1
All neonates	36.7 ± 3.1
Birth Weight (grams)	
Very preterm	1276 ± 488
Near term and term	2900 ± 682
All neonates	2776 ± 803
Duration of hospital admission (days)	
Very preterm	29.4 ± 25.1
Near term and term	10.2 ± 12.3
All neonates	12.7 ± 15.9
Male Gender [Number (%)]	
Very preterm	92 (55.4%)
Near term and term	398 (51.2%)
All neonates	490 (52%)
5 minute Conventional Apgar score	
Very preterm	7.7 ± 1.4
Near term and term	9.4 ± 0.8
All neonates	9.2 ± 1
5 minute Combined Apgar score	
Very preterm	10.1 ± 3.9
Near term and term	15.5 ± 2.5
All neonates	15 ± 3.1

SD: Standard Deviation

### Depressed Conventional-Apgar score at 5-minutes and adverse neonatal outcomes

Depressed 5-minute Conventional-Apgar scores (less than 7) were detected in 50 neonates (5%). Before adjusting for the potential confounders, a depressed 5-minute Conventional-Apgar score was significantly associated with IVH (p<0.001), mechanical ventilation (p<0.001), and neonatal mortality (p<0.001) in the very preterm admitted neonates (Table 4). Similarly, among the near term and term admitted neonates a depressed 5-minute Conventional-Apgar score was significantly associated with IVH (p<0.001), mechanical ventilation (p<0.001), severe jaundice (p<0.001) and neonatal mortality (p = 0.001, Table 4).

**Table 4:** Association between depressed 5- minute Conventional-Apgar score and different neonatal outcomes (total n = 942).

Outcomes	Low 5- minute Conventional-Apgar		Unadjusted OR (95% CI)	Un-adjusted P value	Adjusted OR (95% CI) †	Adjusted P value †
	Yes (n = 50) N (%)	No (n = 892) N (%)				
Mechanical ventilation						
Very Preterm (n = 40)	12 (24%)	28 (3.1%)	9.74 (4.6–20.63)	<0.001	1.23 (0.14–10.71)	0.84
Near term and term (n = 22)	12 (24%)	10 (1.1%)	27.85 (11.32–68.49)	<0.001	0.32 (0.02–4.05)	0.38
All neonates (n = 62)	24 (48%)	38 (4.3%)	20.74 (10.9–39.45)	<0.001	0.82 (0.18–3.76)	0.8
Severe hyperbilirubinemia						
Very Preterm (n = 53)	3 (6%)	50 (5.6%)	1.07 (0.32–3.57)	0.75	0.52 (0.13–2.11)	0.36
Near term and term (n = 126)	17 (34%)	109 (12.2%)	3.7 (1.99–6.86)	<0.001	1.75 (0.48–6.32)	0.39
All neonates (n = 179)	20 (40%)	159 (17.8%)	3.07 (1.7–5.55)	<0.001	1.05 (0.44–2.46)	0.9
IVH						
Very Preterm (n = 33)	12 (24%)	21 (2.4%)	13.09 (6–28.57)	<0.001	0.78 (0.15–4.12)	0.77
Near term and term (n = 14)	5 (10%)	9 (1%)	10.9 (3.5–33.86)	<0.001	0.93 (0.06–13.45)	0.95
All neonates (n = 47)	17 (34%)	30 (3.4%)	14.8 (7.43–29.48)	<0.001	0.7 (0.18–2.75)	0.61
Neonatal mortality						
Early Preterm (n = 19)	10 (20%)	9 (1%)	24.52 (9.44–63.71)	<0.001	0.14 (0.007–2.67)	0.19
Near term and term (n = 5)	3 (6%)	2 (0.2%)	28.4 (4.63–174.09)	0.001	4.25 (0.12–151.13)	0.42
All neonates (n = 24)	13 (26%)	11 (1.2%)	28.14 (11.81–67.01)	<0.001	0.65 (0.11–3.9)	0.64

OR: odds ratio

95% CI: 95% confidence interval

†: adjusted for gestational age, birth weight, and the requirement for resuscitation in the delivery room

IVH: intraventricular hemorrhage

We used a Logistic Regression model to adjust the results for gestational age, birth weight and the requirement for resuscitation in the delivery room; interestingly, after the adjustments, a low 5-minute Conventional-Apgar score lost its significant association with all the studied outcomes (Table 4).

### Depressed Combined-Apgar score at 5 minutes and adverse neonatal outcomes

Low 5-minute Combined-Apgar scores (less than 10) were detected in 95 neonates (10%). Before adjusting for the potential confounders, a depressed 5-minute Combined-Apgar score was significantly associated with IVH (p<0.001), mechanical ventilation (p<0.001), severe jaundice (p<0.001) and neonatal mortality (p<0.001) among the very preterm admitted neonates (Table 5). Similarly, among the near term and term admitted neonates a depressed 5-minute Combined-Apgar score was significantly associated with IVH (p<0.001), mechanical ventilation (p<0.001), severe jaundice (p = 0.02) and neonatal mortality (p<0.001, Table 5).

**Table 5:** Association between depressed 5- minute Combined-Apgar score and different neonatal outcomes (total n = 942).

Outcomes	Low 5- minute Combined-Apgar		Unadjusted OR (95% CI)	Un-adjusted P value	Adjusted OR (95% CI) †	Adjusted P value †
	Yes (n = 95) N (%)	No (n = 847) N (%)				
Mechanical ventilation						
Very Preterm (n = 40)	28 (28.9%)	12 (1.4%)	28.23 (13.75–57.97)	<0.001	24.23 (4.05–144.97)	<0.001
Near term and term (n = 22)	10 (10.5%)	12 (1.4%)	8.18 (3.43–19.5)	<0.001	10.98 (2.44–49.26)	0.002
All neonates (n = 62)	38 (40%)	24 (2.8%)	22.86 (12.83–40.71)	<0.001	18.61 (6.75–51.29)	<0.001
Severe hyperbilirubinemia						
Very Preterm (n = 53)	18 (18.9%)	35 (4.1%)	5.42 (2.93–10.02)	<0.001	6.88 (1.13–41.89)	0.03
Near term and term (n = 126)	20 (21%)	106 (12.5%)	1.86 (1.09–3.17)	0.02	1.75 (0.48–6.32)	0.39
All neonates (n = 179)	38 (40%)	141 (16.6%)	3.33 (2.13–5.22)	<0.001	2.48 (0.74–8.27)	0.13
IVH						
Very Preterm (n = 33)	18 (18.9%)	15 (1.8%)	12.96 (6.28–26.74)	<0.001	7.85 (1.89–32.55)	0.005
Near term and term (n = 14)	8 (8.4%)	6 (0.7%)	12.88 (4.37–38)	<0.001	3.33 (1.03–10.77)	0.04
All neonates (n = 47)	17 (27.4%)	30 (2.5%)	14.82 (7.93–27.69)	<0.001	4.8 (1.91–12.01)	0.001
Neonatal mortality						
Early Preterm (n = 19)	13 (13.7%)	6 (0.7%)	22.22 (8.22–60.01)	<0.001	19.29 (2.31–123.02)	0.009
Near term and term (n = 5)	5 (5.2%)	0 (0%)	-	<0.001	-	<0.001
All neonates (n = 24)	18 (18.9%)	11 (0.7%)	32.76 (12.63–84.97)	<0.001	20.22 (4.22–96.88)	<0.001

OR: odds ratio

95% CI: 95% confidence interval

†: adjusted for gestational age, birth weight, and the requirement for resuscitation in the delivery room

IVH: intraventricular hemorrhage

We used a Logistic Regression model to adjust the results for gestational age, birth weight and the requirement for resuscitation in the delivery room; after the adjustments among the very preterm admitted neonates, a depressed 5-minute Combined-Apgar score remained significantly associated with the requirement for mechanical ventilation (p<0.001), IVH (p = 0.005), neonatal mortality (p = 0.009), and severe hyperbilirubinemia (p = 0.03, Table 5). Similarly, after the adjustments in the near term and term admitted neonates, a depressed 5-minute Combined-Apgar score remained significantly associated with the requirement for mechanical ventilation (p = 0.002), IVH (p = 0.04), and neonatal mortality (p<0.001) but not with severe hyperbilirubinemia (p = 0.39, Table 5).

### Comparison between Conventional versus Combined Apgar scores in predicting neonatal mortality and morbidity

The predictive values of the Conventional and Combined scores were compared by calculating the areas under the curve (AUC) and their co-variances of the ROC for the occurrence of neonatal mortality, IVH, severe jaundice and the requirement for mechanical ventilation (Table 6). The AUC was higher for the Combined-Apgar score than the Conventional-Apgar score in predicting neonatal mortality, IVH, requirement for mechanical ventilation and severe jaundice among all the admitted neonates and their subgroups (Table 6).

**Table 6:** Comparison of the area under the curve (AUC) of the ROC Curve for the combined versus conventional Apgar scores.

Outcome	Conventional Apgar		Combined Apgar	
	AUC (95%CI)	P value	AUC (95%CI)	P value
Mechanical ventilation				
Very preterm	0.7 (0.56–0.84)	0.009	0.92 (0.85–0.99)	<0.001
Near term and term	0.78 (0.67–0.89)	<0.001	0.94 (0.9–0.98)	<0.001
All neonates	0.84 (0.78–0.91)	<0.001	0.96 (0.93–0.98)	<0.001
Severe hyperbilirubinemia				
Very preterm	0.59 (0.44–0.74)	0.21	0.72 (0.58–0.85)	0.006
Near term and term	0.49 (0.42–0.57)	0.97	0.52 (0.44–0.6)	0.51
All neonates	0.52 (0.5–0.63)	0.02	0.58 (0.51–0.65)	0.02
IVH				
Very preterm	0.58 (0.42–0.74)	0.28	0.78 (0.65–0.9)	<0.001
Near term and term	0.69 (0.57–0.8)	0.004	0.77 (0.66–0.88)	<0.001
All neonates	0.75 (0.67–0.83)	<0.001	0.83 (0.76–0.9)	<0.001
Neonatal death				
Very preterm	0.69 (0.55–0.84)	0.02	0.92 (0.85–0.99)	<0.001
Near term and term	0.87 (0.76–0.98)	0.001	0.97 (0.95–0.98)	<0.001
All neonates	0.90 (0.86–0.96)	<0.001	0.97 (0.96–0.99)	<0.001

ROC: Receiver Operating Characteristic

95%CI: 95% Confidence Interval

IVH: Intraventricular Hemorrhage

## Discussion

From shortly after its introduction in the 1950s until now, the Conventional-Apgar score has been used by numerous studies for predicting adverse neonatal outcomes.[2] Because the initial purpose of the Apgar score was assessing the newborn's condition in the delivery room rather than predicting neonatal outcomes, later, Virginia Apgar warned that her score could be used to predict outcomes in individual infants but only in groups of infants.[15] The ACOG and AAP in their 2006 and 2015 committee opinions emphasized that, due to its limitations, the Apgar score should not be used alone to predict adverse neonatal outcomes.[9,16] The main limitation of the Apgar score is that it is influenced by the gestational age, neonatal maturity, drugs, trauma, hypoxemia, hypovolemia and the interventions in the delivery room; thus an Apgar score assigned during resuscitation and clinical interventions does not give a precise assessment of the newborn's situation.[6,9,16,17] Additionally, the Conventional-Apgar score has been shown to have poor reproducibility and inter-observer reliability in the individual newborn.[6,8,16,17]

To overcome the problem of reproducibility of the Conventional-Apgar, the Specified-Apgar score was introduced by Rudiger et al. using the same items as the Conventional-Apgar with more detailed and strict definitions for the newborn’s condition regardless of gestational age and interventions.[10] To overcome the limitations of the medical interventions required to achieve this condition, the Expanded-Apgar score was described by the AAP and ACOG, consisting of 7 items, clearly defining the medical interventions in the delivery room.[9] With the improvement of neonatal care and the increase in the survival of the resuscitated and premature neonates, an assessment tool was required to represent both the medical interventions and the neonatal condition; therefore Rudiger and Aguar introduced the Combined-Apgar score, which combines both the Specified and Expanded Apgar scores and ranges from 0 to 17 points.[12] A score of 17 describes the perfect clinical condition of an infant (Specified-Apgar score of 10) without any medical intervention (Expanded-Apgar score of 7). In contrast, a score of 0 describes the poor clinical condition of an infant who has received all resuscitative interventions without any clinical response.[12]

In the current study, although before adjustments, a depressed 5-minute Conventional-Apgar score was associated with neonatal mortality, after adjustment for gestational age, birth weight, and neonatal resuscitation, these associations failed to reach the significance level. Literature on the predictive value of the Conventional-Apgar score for neonatal mortality has conflicting results; some studies showed a limited value,[18,19] whereas others indicated a continuing value for the Conventional-Apgar score in predicting neonatal death.[3] Importantly, none of the previous studies assessed had adjusted the results for the potential confounders.[4] Iliodromiti et al., in their study of over one million neonates, adjusted the results for the important confounders including gestational age and birth weight.[4] They documented that a low 5-minute Conventional-Apgar score was associated with an increased risk of neonatal death. However, the strength of the association was strongest at term.[4] They also indicated that in premature infants, a low Conventional-Apgar score does not necessarily reflect a poor neonatal condition, but it could be due to intrinsic physiological immaturity and an inadequate capacity for response; therefore the association of the depressed Conventional-Apgar score and infant death is attenuated in prematurity.[4]

In our study we assessed for the first time the value of the Combined-Apgar score in predicting neonatal mortality among all the admitted neonates including near term and term neonates. Compared to the Conventional-Apgar score, the newly proposed Combined-Apgar score was a better predictor of neonatal mortality among all admitted neonates and also in the subgroups of very preterm, near term and term neonates; this finding was independent of gestational age, birth weight and neonatal resuscitation in the delivery room. In our study, a low 5-minute Combined-Apgar score was associated with an almost 20-fold increased risk for neonatal mortality in very preterm neonates and a 15-fold increased risk for neonatal death in near term and term neonates. Rudiger et al. tested the value of the Combined-Apgar score in predicting neonatal mortality in a large cohort of very preterm neonates. In their study, a very low Combined-Apgar score was associated with a 30-fold increased risk for perinatal mortality in very preterm neonates.[13]

In this study, a depressed 5-minute Combined-Apgar score was independently associated with IVH and the requirement for mechanical ventilation in all admitted neonates and their gestational age subgroups. We also showed that the Combined-Apgar score could predict the adverse neonatal outcomes better than the Conventional-Apgar score in very preterm, near term and term admitted neonates. This was in accordance with two other studies that were conducted on very preterm infants[13] and asphyxiated infants.[14] Previously we had shown that the Combined-Apgar score has the highest sensitivity and specificity among the proposed scores (Conventional, Specified, and Expanded Apgar scores) in predicting birth asphyxia and the occurrence of IVH in asphyxiated neonates.[14] Also, Rudiger et al. illustrated that the Combined-Apgar score was a better predictor of poor neonatal outcomes, including IVH and bronchopulmonary dysplasia in very preterm neonates.[13] However, our study was the first to show that the low Combined-Apgar score increases the risk of IVH not only in very preterm neonates but also in near term and term neonates.

The newly proposed Combined score allows a more appropriate description of the infant’s condition under conditions of modern neonatal care.[13] Very premature neonates who might previously have died now have increased survival, and many infants now receive medical interventions during the first minutes of life. Therefore, due to the changes in the care of the newborns during the past 60 years, the Conventional-Apgar score seems to have poor reliability as an outcome measure at the present time, especially for the preterm and resuscitated neonates.[2,4,6,9,13,14,20] However, it should be emphasized that the Combined-Apgar score was never intended to replace but rather to specify the Conventional-Apgar score.[13] Therefore, the items of the Conventional score were neither changed nor omitted.[12,13]

This study tested for the first time the applicability of the newly proposed Combined-Apgar score in a large group of admitted very preterm, near term and term infants; it is among the very first studies to use the Combined-Apgar score in clinical practice and to compare its predictive value to the Conventional-Apgar score in predicting adverse neonatal outcomes. Further prospective studies with larger sample sizes are required to confirm these results. Also, studies are needed to test the reproducibility and inter-observer reliability of the Combined-Apgar scoring system. Additionally, long-term follow up studies are required to assess if a depressed Combined-Apgar score is associated with any long-term disabilities.
